# An Essential Role for the Proximal but Not the Distal Cytoplasmic Tail of Glycoprotein M in Murid Herpesvirus 4 Infection

**DOI:** 10.1371/journal.pone.0002131

**Published:** 2008-05-07

**Authors:** Janet S. May, Christopher M. Smith, Michael B. Gill, Philip G. Stevenson

**Affiliations:** Division of Virology, Department of Pathology, University of Cambridge, Cambridge, United Kingdom; Institut Pasteur Korea, Republic of Korea

## Abstract

Murid herpesvirus-4 (MuHV-4) provides a tractable model with which to define common, conserved features of gamma-herpesvirus biology. The multi-membrane spanning glycoprotein M (gM) is one of only 4 glycoproteins that are essential for MuHV-4 lytic replication. gM binds to gN and is thought to function mainly secondary envelopment and virion egress, for which several predicted trafficking motifs in its C-terminal cytoplasmic tail could be important. We tested the contribution of the gM cytoplasmic tail to MuHV-4 lytic replication by making recombinant viruses with varying C-terminal deletions. Removing an acidic cluster and a distal YXXΦ motif altered the capsid distribution somewhat in infected cells but had little effect on virus replication, either *in vitro* or *in vivo*. In contrast, removing a proximal YXXΦ motif as well completely prevented productive replication. gM was still expressed, but unlike its longer forms showed only limited colocalization with co-transfected gN, and in the context of whole virus appeared to support gN expression less well. We conclude that some elements of the gM cytoplasmic tail are dispensible for MuHV-4 replication, but the tail as a whole is not.

## Introduction

Herpesviruses lytic gene functions have been studied mainly with neurotropic alpha-herpesviruses. Relatively little is known about the lytic replication of lymphotropic gamma-herpesviruses. This reflects that Epstein-Barr virus (EBV) and the Kaposi's Sarcoma-associated Herpesvirus (KSHV) - the two known human gamma-herpesviruses - are difficult to propagate lytically *in vitro* and have narrow species tropisms that largely preclude their analysis *in vivo*. However, the gamma-herpesviruses of other animals offer tractable approaches to what are fundamentally the same biological questions: how are new virions formed and how do they spread.

A well-established small animal model of gamma-herpesvirus infection is provided by Murid Herpesvirus-4 (MuHV-4), a rhadinovirus closely related to KSHV [1], [2]. MuHV-4 is B cell tropic like EBV and KSHV [3], but also propagates lytically in a range of fibroblast and epithelial lines. The main natural host for MuHV-4 is yellow-necked mice [4]. It appears to have quite a broad tropism [1] and also behaves much like a natural pathogen in inbred laboratory mouse strains [5], [6]. Thus, intranasal infection causes an infectious mononucleosis-like illness followed by asymptomatic viral persistence [7]; and immune suppression causes disease [8]. Some 90% of MuHV-4 genes have obvious equivalents in KSHV and EBV [9]. This is particularly true of lytic genes; latency-associated genes are less well conserved. MuHV-4 is therefore perhaps best suited to analyzing gamma-herpesvirus lytic functions. Our interest is in the virion glycoproteins. The complement of essential MuHV-4 glycoproteins is surprisingly small: only gH, gB, gN and gM [10]–[12] are essential; gp70 [13], [14], gp48 [15], ORF28 [16], ORF58 [17], gL [18], gp150 [19] and ORF74 [20], [21] are not.

gM is among the most conserved of all herpesvirus glycoproteins. It has 8 transmembrane domains without a large extracellular domain, forms a disulfide-linked complex with gN [12], [22]–[24], and functions mainly in virion assembly and egress [25]–[27]. Consistent with this role, co-transfected gM and gN co-localize in the trans-Golgi network [12], [23], [28] where herpesvirus secondary envelopment occurs [29]. The gM cytoplasmic tail contains several trafficking motifs whose conservation in alpha-, beta- and gamma-herpesviruses suggests that they have important functions. These include YxxΦ motifs [30] which have been linked to AP2 binding [31], and an acidic cluster which may be involved in trans-Golgi targetting [32].

Herpesviruses vary surprisingly widely in their requirement for gM [33]–[36]. In order to understand better the function of the MuHV-4 gM, we have addressed specifically the role of its cytoplasmic tail by generating recombinant viruses with cytoplasmic tail deletions. The distal cytoplasmic tail, including 2 conserved trafficking motifs, was dispensible for lytic replication, both *in vitro* and *in vivo*. Yet the tail as a whole was not. Thus, the gM transmembrane domains alone were insufficient to support MuHV-4 replication.

## Materials and Methods

### Mice

Female C57BL/6 mice were purchased from Harlan U.K. Ltd. (Bicester, U.K.), housed in the Cambridge University Department of Pathology, and infected intranasally with MuHV-4 (10^4^ p.f.u.) when 6–8 weeks old. Animal welfare conformed to the UK Animal Health Act of 1981 (and subsequent amendments). All animal experiments were performed under and in accordance with Home Office Project Licence 80/1992.

### Cell lines

BHK-21 fibroblasts, 293T cells, NIH-3T3-fibroblasts, the cre recombinase-expressing derivative 3T3-CRE [37], and murine embryonic fibroblasts (MEFs) were grown in Dulbecco's modified Eagle medium (Invitrogen, Paisley, U.K.) supplemented with 2 mM glutamine, 100 U/ml penicillin, 100 µg/ml streptomycin and 10% fetal calf serum (PAA laboratories, Linz, Austria). Medium for MEFs was further supplemented with 50 µM 2-mercaptoethanol. Cells were transfected where indicated using Fugene-6 (Roche Diagnostics, Ltd., Lewes, U.K.).

### Viral mutagenesis

The gM coding sequence without its stop codon (genomic co-ordinates 56950–55796, 383 amino acids) [9] was cloned as a *Bam*HI/*Xho*I-restricted PCR product into the *Bam*HI/*Xho*I sites of pEGFP-N3 (Clontech, Palo Alto, CA). Genomic co-ordinates 55800–54649 were then cloned into the *Afl*II/*Not*I sites of the same vector to provide a second recombination flank. Variants of this full-length form (383 amino acid residues) were generated by shifting the *Bam*HI-restricted PCR primer further 5′ in the gM coding sequence. Thus, we deleted amino acid residues 378–383 (T4), 365–383 (T3), 352–383 (T2) or 337–383 (T1). Each shorter PCR product was exchanged for the full-length form by digesting the PCR product and gM-eGFP vector with *Bam*HI and *Xho*I, removing the full-length gM insert, and ligating the fragments together. Each gM mutant was thus fused to eGFP at a *Bam*HI restriction site (encoding glycine+serine). We also made versions of the T2, T3 and T4 truncations in which eGFP was separate to gM, by inserting the self-complementary oligonucleotide 5′-CTAGCTAGCTAGAATTCTAGCTAGCTAG-3′, which includes multiple stop codons, into the *Bam*HI site, for this purpose blunted with Klenow fragment DNA polymerase and dephosphorylated with Antarctic alkaline phosphatase (New England Biolabs, Hitchin, U.K.). Each construct was subcloned into the *Bam*HI/*Sac*I sites of the pST76K-SR shuttle vector using a *Bgl*II site in the pEGFP-N3 polylinker and a *Sac*I site incorporated into the *Afl*II flank primer. Each was then recombined into the MuHV-4 BAC by standard methods [13]. Where indicated, we also derived revertant BACs in which the mutant locus was replaced by the native genomic sequence. Infectious virus was reconstituted (or not) by transfecting BAC DNA into BHK-21 cells. Except where indicated, the loxP-flanked BAC/eGFP cassette was removed from viable viruses by passage through 3T3-CRE cells. Virus stocks were grown in BHK-21 cells. Infected cells plus supernatants were freeze-thawed when most cells showed cytopathic effects (4–5 days post inoculation with 0.001 p.f.u./cell). Cell debris was pelleted by low-speed centrifugation (1000×*g*, 3 min) and discarded. Virions were then recovered from supernatants by high speed centrifugation (38,000×*g*, 90 min) and stored at −70°C.

### Virus titrations

Infectious virus was titered by plaque assay on BHK-21 cells [38]. Briefly, 10-fold virus dilutions were incubated on cell monolayers (2 h, 37°C) then overlaid with 0.3% carboxymethylcellulose. The monolayers were fixed in 4% formaldehyde 4 days later and stained with 0.1% toluidine blue. Plaques were counted with a plate microscope. To titer infectious virus in lungs, the lungs were homogenized in complete medium, then frozen and thawed. Tissue debris was pelleted by brief centrifugation (1000×*g*, 1 min) and homogenate supernatants were titered by plaque assay. Latent virus in spleens was measured by infectious center assay [38]. Single spleen cell suspensions were cultured on MEF monolayers, again overlaid with 0.3% carboxymethylcellulose, which were fixed and stained after 5 days. Pre-formed infectious virus - that forming plaques after freeze-thawing of the spleen cells - always contributed <1% of the infectivity recoverable from lymphoid tissue, so the infectious center assay essentially measured reactivatable latent virus.

### Southern blotting

Viral DNA was extracted by alkaline lysis [38], digested with restriction endonucleases, electrophoresed through 0.8% agarose in Tris acetate buffer and transferred to positively charged nylon membranes (Roche Diagnostics). A ^32^P-dCTP labelled probe (APBiotech, Little Chalfont, U.K.) was generated by random primer extension (Nonaprimer kit, Qbiogene, Bingham, U.K.) using the *Bam*HI-B genomic clone (49938–59884) as a template [39]. The membranes were hybridised with the probe (65°C, 18 h), washed to a stringency of 0.2% SSC, 0.1% SDS, and then exposed to X-ray film.

### Immunofluorescence and confocal microscopy

Adherent cells (BHK-21, 293T or NIH-3T3) were washed in PBS, fixed in 2% paraformaldehyde or 100% methanol, then permeabilized with 0.1% Triton-X100. The MuHV-4 gN was detected with mAb 3F7 [12], the ORF65 capsid component with mAb MG-12B8 [40] and thymidine kinase with mAb CS-4A5. MAb CS-4A5 was derived from MuHV-4 carrier mice and identified as thymidine kinase-specific by staining 293T cells transfected or not with a MuHV-4 ORF21 expression plasmid. Its specificity was confirmed by recognizing BHK-21 cells infected with wild-type but not thymidine kinase-deficient mutants [38]. Bound mAb was detected with Alexa 568-conjugated goat anti-mouse IgG pAb (Invitrogen Corporation, Paisley, U.K.). The cells were washed ×2 in PBS/0.1% Tween-20 after each antibody incubation. Nuclei were counterstained with DAPI. Fluorescence was visualized with an Olympus IX70 microscope plus a Retiga 2000R camera line (QImaging) or with a Leica confocal microscope.

## Results

### Generation of gM C-terminal truncation BAC mutants

**Figure 1:**
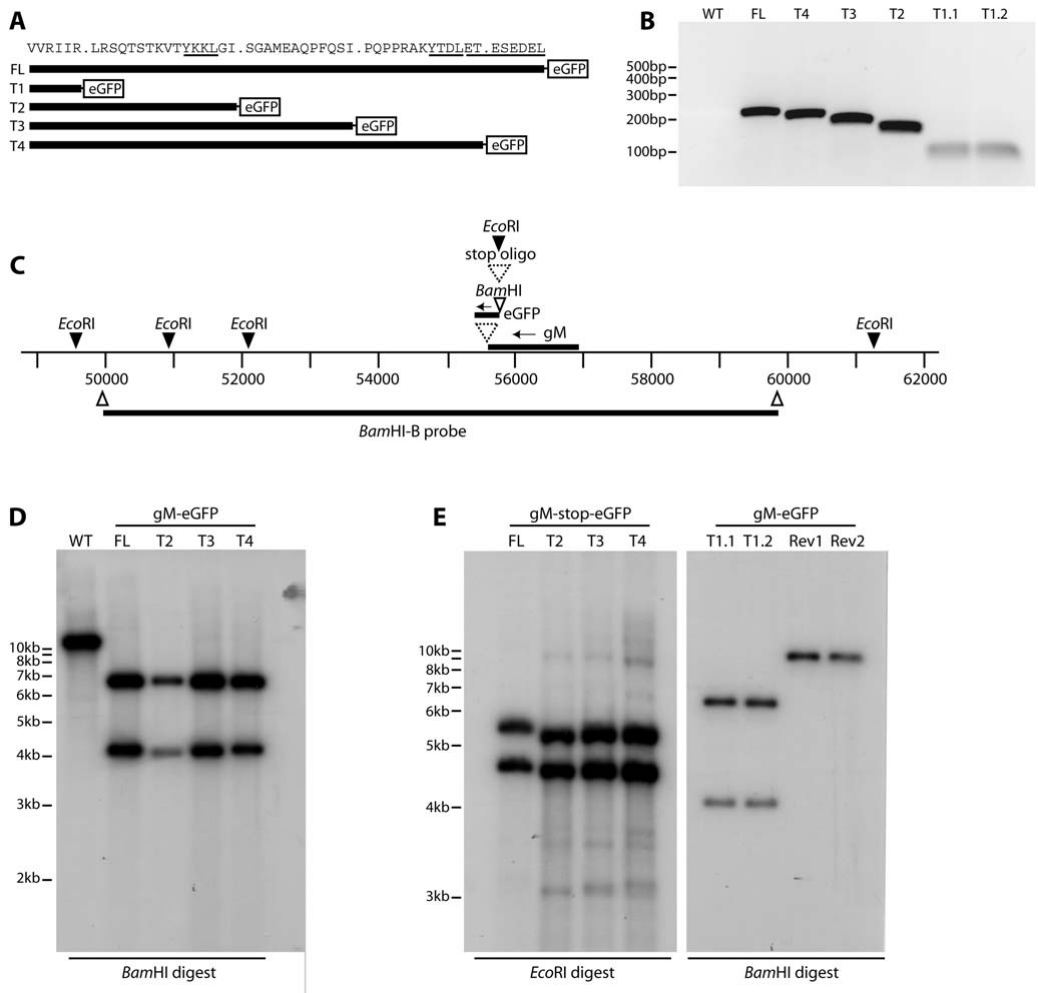
Generation of MuHV-4 gM cytoplasmic tail truncation mutants. A. The MuHV-4 gM cytoplasmic tail contains 2 YxxΦ motifs and an acidic cluster. Truncation mutants were designed to delete sequentially each motif, as well as a further deletion of the amino acid residues between the 2 YxxΦ motifs. Each gM derivative was tagged with eGFP to allow its visualization in infected cells. B. Because the truncations were too small to be distinguished by Southern blotting, we checked them by PCR, using a 5′ gM primer just upstream of the deletions (genomic co-ordinates 55944–55962) and a 3′ eGFP primer 65 nt from its start of codon. WT = wild-type, which does not amplify because it has no eGFP tag on gM. FL = full-length tagged gM. T1.1 and T1.2 are independent mutants. The correctness of each deletion was confirmed by DNA sequencing its PCR product. C. The MuHV-4 gM locus was modified by inserting a C-terminal eGFP tag. On this scale, the truncations of each mutant are too small to discern. Independent FL T2, T3 and T4 mutants were made with an inserted oligonucleotide terminating translation before the eGFP tag. D. Viral DNA from wild-type MuHV-4 (WT) or eGFP-tagged gM derivatives (FL, T2, T3, T4) was digested with *Bam*HI, electrophoresed, blotted and probed with the *Bam*-B genomic fragment as shown in C. Inserting the eGFP coding sequence with its 5′ *Bam*HI site converted a 10 kb band to 4.1 kb+6.6 kb (eGFP contributing 0.7 kb). The T1 mutant was not included in this blot because it did not reconstitute infectious virus. E. DNA was recovered from each untagged gM mutant virus. The oligonucleotide separating gM from the eGFP tag has a diagnostic *Eco*RI restriction site. The viral DNA was digested with *Eco*RI and probed across the *Bam*HI-B region as before. Oligonucleotide insertion converted a 9.2 kb *Eco*RI-restricted band to 5.5 kb+4.4 kb. The difference in migration between the FL and T2–T4 viruses reflects a difference in DNA preparation rather than a real difference in size, since slightly slower migration was observed for all the FL bands on ethidium bromide staining (data not shown). The small *Eco*RI bands at genomic co-ordinates 50,000–52,000 are not visible on this gel. BAC DNA from the T1.1 and T1.2 independent mutants was analyzed at the same time by *Bam*HI digestion and *Bam*HI-B probing. Rev1 and Rev2 are revertant BACs for each mutant.

We have previously used C-terminal eGFP tagging to visualize the MuHV-4 gM in infected cells [40]–[42]. We made 4 gM truncation mutants with the same eGFP tag. The truncations were designed to remove progressively a C-terminal acidic cluster (gM-T4), a distal YXXΦ motif (gM-T3), an intervening region (gM-T2) and a proximal YXXΦ motif (gM-T1) (Fig. 1A). PCR of BAC DNA, from just upstream of the deletion site to the 5′ end of the eGFP coding sequence, confirmed the predicted size of each gM cytoplasmic tail (Fig. 1B). The region around the gM locus (Fig. 1C) was further checked by Southern blot of viral DNA (Fig. 1D, 1E). Because the T1 truncation mutant failed to reconstitute infectious virus (see below), we analyzed it as BAC DNA, comparing 2 independently derived mutants to their revertants. We also made versions of the FL (full-length), T2, T3 and T4 (truncated) gM-eGFP fusions in which stop codons separated the gM C-terminus from its eGFP tag (Fig. 1E), so as to control for any effect of eGFP tagging.

### Reconstitution of infectious virus from BAC DNA

**Figure 2:**
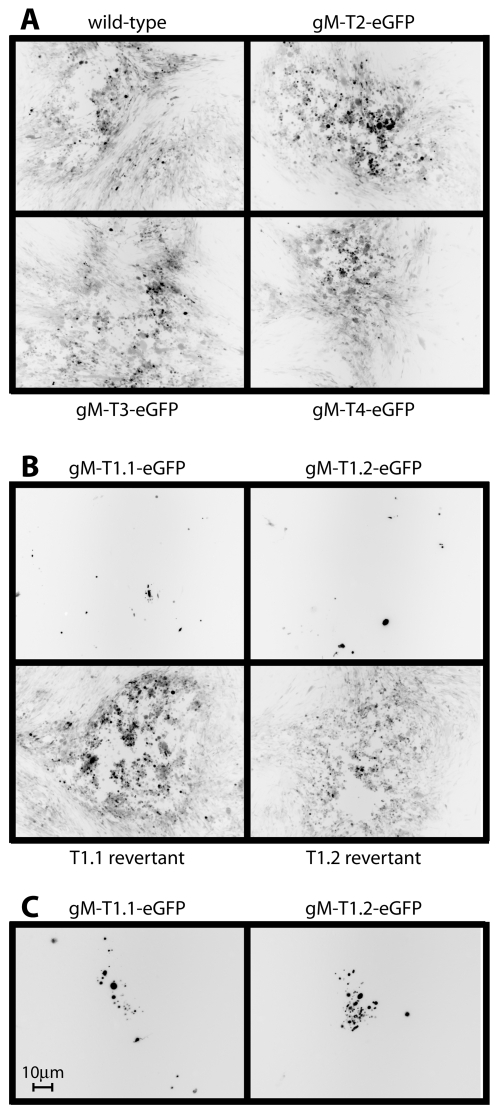
Viral spread visualized by eGFP expression following BAC DNA transfection. A. BHK-21 cells were transfected with wild-type or gM truncation mutant BAC DNA. 72 h later, infection was monitored by fluorescence microscopy. Each virus expresses eGFP from an HCMV IE-1 promoter in addition to the eGFP tag on gM. Cells expressing virus-encoded eGFP appear black. B. Two independent gM-T1 mutants were compared with their revertants in an experiment similar to A. The images shown are from 72 h post-transfection. There was also no sign of virus replication at 6 days post-infection. C. Higher magnification images of BHK-21 cells after T1.1 and T1.2 BAC transfection showed small clusters of eGFP^+^ fragments. But these were smaller than BHK-21 cells (the scale bar shows 10 µm), and were presumably non-infectious debris derived from apoptotic BAC-transfected cells. There was no sign of the neighbouring BHK-21 cells becoming infected.

After transfecting BAC DNA into BHK-21 cells, the propagation of the T2, T3 and T4 gM mutants was indistinguishable from that of the wild-type (Fig. 2A). In contrast, there was no spread of eGFP signal with either of 2 independently-derived T1 mutants (Fig. 2B). Nor was there recoverable T1 mutant infectivity by plaque assay (data not shown). Higher magnification images of cells transfected with each T1 mutant BAC (Fig. 2C) showed small clusters of eGFP^+^ debris, but no sign of infection spread. Thus, the distal YXXΦ motif and the acidic cluster of the gM cytoplasmic tail were redundant for *in vitro* MuHV-4 replication, but the tail as a whole was not.

### Viral protein expression by the non-viable gM truncation mutant

**Figure 3:**
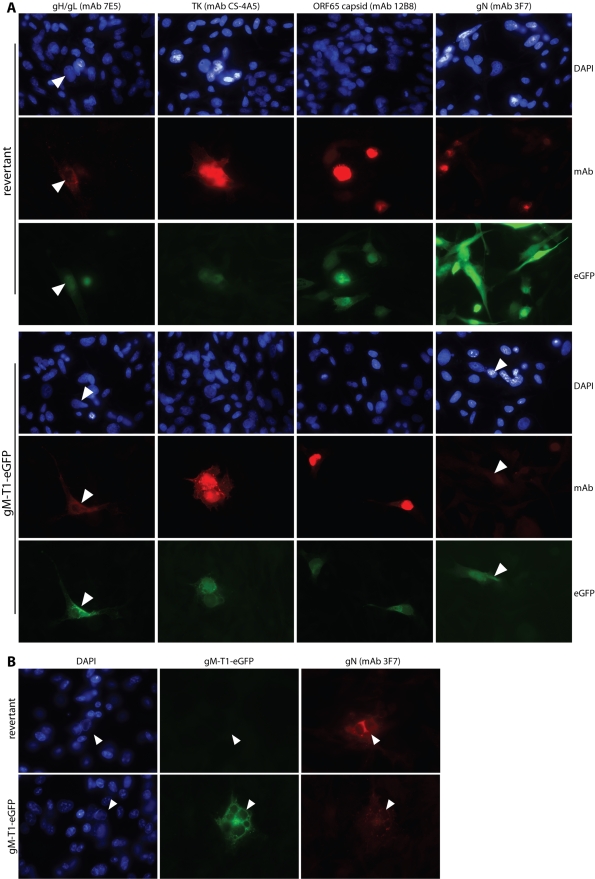
Viral protein expression by MuHV-4 lacking its gM cytoplasmic tail. A. BHK-21 cells were transfected with gM-T1 mutant BAC DNA (gM-T1-eGFP) or with the corresponding revertant. 48 h later, the cells were fixed with 2% paraformaldehyde and stained with MuHV-4-specific mAbs plus Alexa 568-conjugated goat anti-mouse IgG pAb (red). eGFP expression - HCMV IE1-driven eGFP alone for the revertant and HCMV IE1-driven eGFP+gM-T1-eGFP for the mutant - was visualized directly (green). Nuclei were stained with DAPI (blue). The arrowheads show transfected cells. B. BHK-21 cells transfected with gM-T1 mutant BAC DNA were fixed instead with 100% methanol. This allowed free eGFP to be washed out, making eGFP fluorescence more specific to gM-T1 expression. The cells were then stained for gN with mAb 3F7, which works equally well with either fixation. Representative images are shown. Similar results were obtained in 2 repeat experiments.

In order to understand why the gM-T1 mutant failed to produce infectious virus, we examined by immunofluorescence BHK-21 cells transfected with gM-T1 BAC DNA (Fig. 3). gH/gL, thymidine kinase and ORF65 (capsid) expression were normal. In contrast, gN expression - which depends on gM [12] - appeared to be lower than for the revertant. Since the gM-T1 mutant did not spread, this analysis was limited by not knowing that that the BAC DNA taken up by a given cell was intact. Also, as BAC-derived viruses express eGFP from a Human cytomegalovirus (HCMV) IE1 promoter [13] independent of endogenous lytic antigens until the BAC cassette is removed [41], [42] (so in Fig. 3A not all the eGFP^+^ cells infected with the revertant BAC express gN) it was difficult to be sure that eGFP^+^ cells transfected with the gM-T1 mutant were really low for gN, or just not supporting lytic infection.

We were able to address this problem by exploiting the eGFP tag on gM. In contrast to the mainly nuclear fluorescence of the free eGFP expressed from the HCMV IE1 promoter, gM-eGFP fluorescence is mainly perinuclear. Thus, Fig. 3A shows subtle differences in intracellular eGFP distribution between the gM-T1 (BAC^+^gM-eGFP^+^) and revertant (BAC^+^gM-eGFP^−^) BACs, with the net distribution eGFP fluorescence after gM-T1 BAC transfection depending on the relative activities of the HCMV IE1 and gM promoters. In order to identify more precisely the gM-T1 BAC-transfected cells supporting lytic infection, we fixed them with methanol rather than paraformaldehyde (Fig. 3B). This retains membrane-bound eGFP, while free eGFP is washed out, so detectable eGFP expression is confined to lytic infection. Using this approach, gN expression by the gM-T1 mutant in eGFP^+^ cells, although detectable, was again weak. Thus, the gM-T1 BAC was capable of lytic gene expression, but appeared to support only limited gN expression.

### Localization of gM-T1-eGFP co-transfected with gN

**Figure 4:**
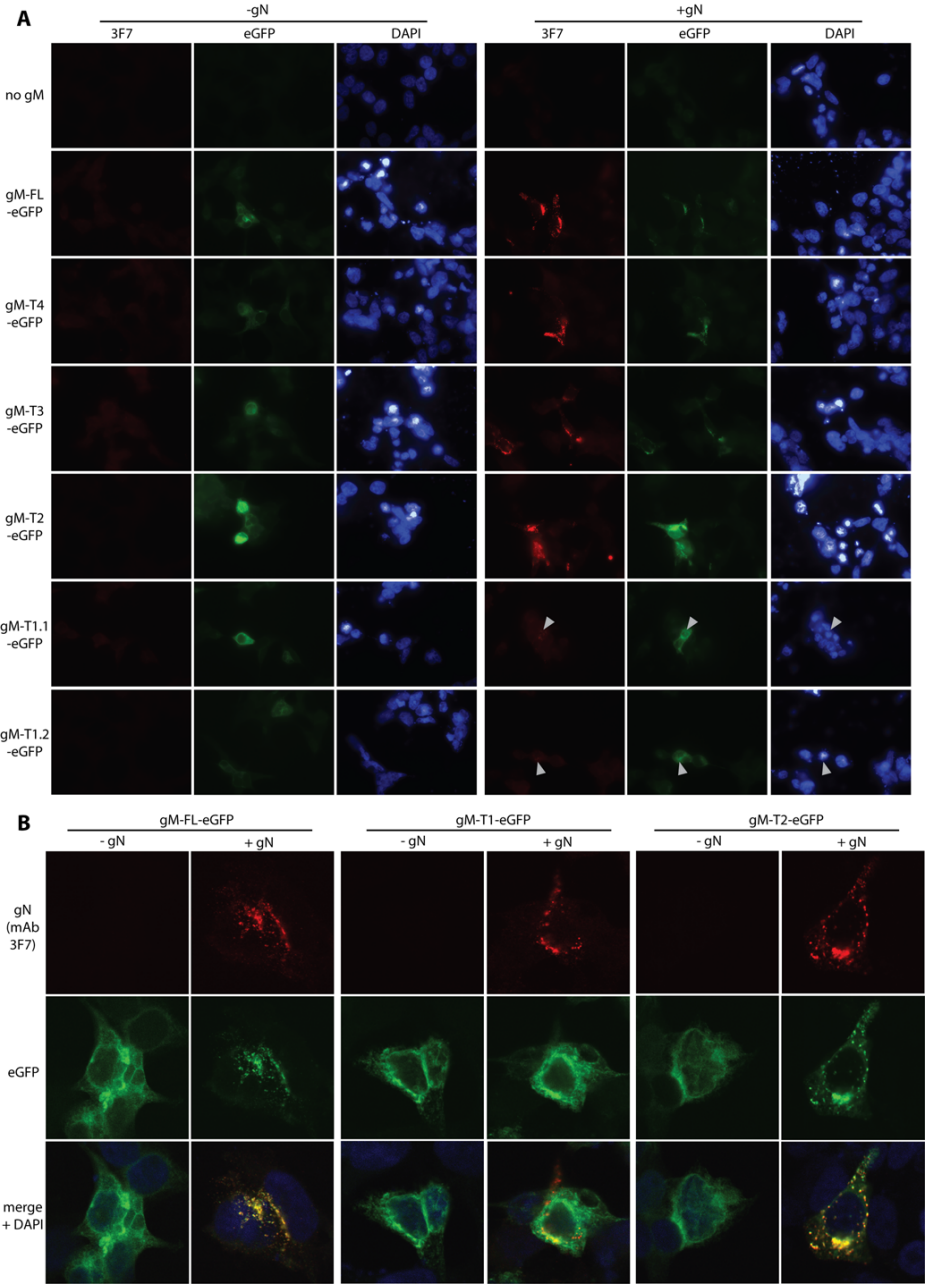
Co-transfection of gM truncation mutants with gN. A. Each gM-eGFP mutant (T1–T4) or the corresponding full-length form (gM-FL-eGFP) was transfected into 293T cells with or without co-transfected gN. We tested 2 independent gM-T1-eGFP plasmid preparations (1.1, 1.2) to allow for possible poor transfection efficiency. At 48 h post-transfection, the cells were fixed in 2% paraformaldehyde and stained for gN with mAb 3F7 plus Alexa 568-conjugated goat anti-mouse IgG pAb red). EGFP fluorescence was visualized directly (green). Nuclei were counter-stained with DAPI (blue). Representative transfected cells are shown. gN was never visible without co-transfected gM. The data are from 1 of 3 equivalent experiments. B. Higher magnification confocal images made it clear that full-length gM (green) was redistributed when gN (red) was present and that their distributions co-localized almost completely (yellow). The same is seen for the second shortest gM truncation (gM-T2). In contrast, gM-T1 - here, for clarity, a cell with atypically high gN expression has been selected - did not obviously change its distribution when gN was supplied, and their co-localization was much less.

Since only limited numbers of BHK-21 cells could be transfected with the 150 kb BAC plasmid, we also examined the effect of the gM-T1 truncation on gN by co-transfecting the corresponding expression plasmids into 293T cells (Fig. 4). Transfected gN could not be identified unless gM was also transfected (Fig. 4A). As the 3F7 epitope on gN is conformation-independent [12], this probably reflects poor gN stability rather than misfolding. Again, gM-T1 co-expression allowed gN detection, but less well than with the longer forms of gM. Thus, the results with expression plasmids were consistent with the weak gN staining after gM-T1 BAC transfection (Fig. 3).

Although each gM truncation could be expressed alone, gN clearly affected the distribution of gM in co-transfected cells. Thus, gN expression changed full-length gM-eGFP from a diffusely perinuclear distribution to one sharply focussed on just one side of the nucleus (Fig. 4A, 4B). This pattern was consistent with gM/gN localizing to the trans-Golgi network [12]. It applied also to the T2, T3 and T4 mutants, but gM-T1, while again perinuclear without gN, did not appreciably change its distribution when gN was co-transfected (Fig. 4B). Thus, gN and gM-T1 showed only limited colocalization, whereas the the other forms of gM co-localized with gN almost completely. The T1 truncation therefore appeared to impair the association of gM with gN.

### Viral protein expression by the viable gM truncation mutants

**Figure 5:**
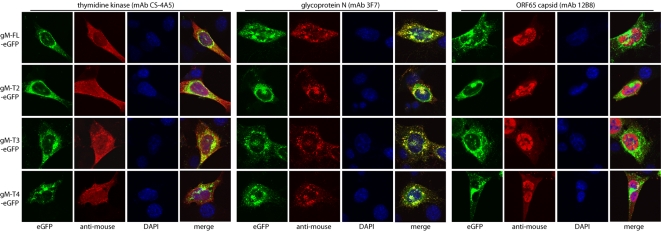
Viral protein distribution in cells infected with viable gM cytoplasmic tail truncation mutants. NIH-3T3 cells were infected with with full-length (FL) or truncated gM-eGFP virus mutants (T2–T4) as shown (0.3 p.f.u./cell, 18 h), then fixed in 2% paraformaldehyde and stained for thymidine kinase, gN or the ORF65 capsid component (red). EGFP fluorescence was visualized directly (green). Co-localization is yellow in the merged image. All these viruses had their BAC-based eGFP expression cassettes removed by loxP recombination, so the only eGFP expressed was that fused to gM. Nuclei were counter-stained with DAPI (blue). The data are from 1 of 3 equivalent experiments.

We next looked at viral protein distribution in cells infected with the viable (T2–T4) gM truncation mutants (Fig. 5). In this context there was again no sign of gM or gN expression being affected by the T2–T4 truncations. Nor was the distribution of thymidine kinase - chosen as a representative tegument protein [43] - obviously different between the mutant viruses and the wild-type. The only consistent abnormality of the mutants was a subtle change in capsid distribution, as revealed by staining for ORF65. Capsid staining was always predominantly nuclear, but each truncation mutant showed additional cytoplasmic capsid staining above that seen with full-length gM.

The predominance of nuclear capsid staining reflects in part that cytoplasmic capsid transport, secondary envelopment and virion egress are all rapid [29]. But another important factor is that capsid antigens are poorly accessible on intact virions because they are masked by the tegument and glycoproteins. This is especially true of the MG-12B8 epitope [40]. The cytoplasmic MG-12B8 staining seen with the T2, T3 and T4 gM mutants therefore suggested that free capsids - or at least capsid components - accumulated in the cytoplasm. Thus, the immunofluorescence pictures were consistent a delay in secondary envelopment. The lack of an obvious difference between the different truncation mutants implied that this phenotype was due to disruption of the acidic cluster at the very C-terminus of the gM cytoplasmic tail (Fig. 1A).

### *In vitro* growth of the viable gM truncation mutants

**Figure 6:**
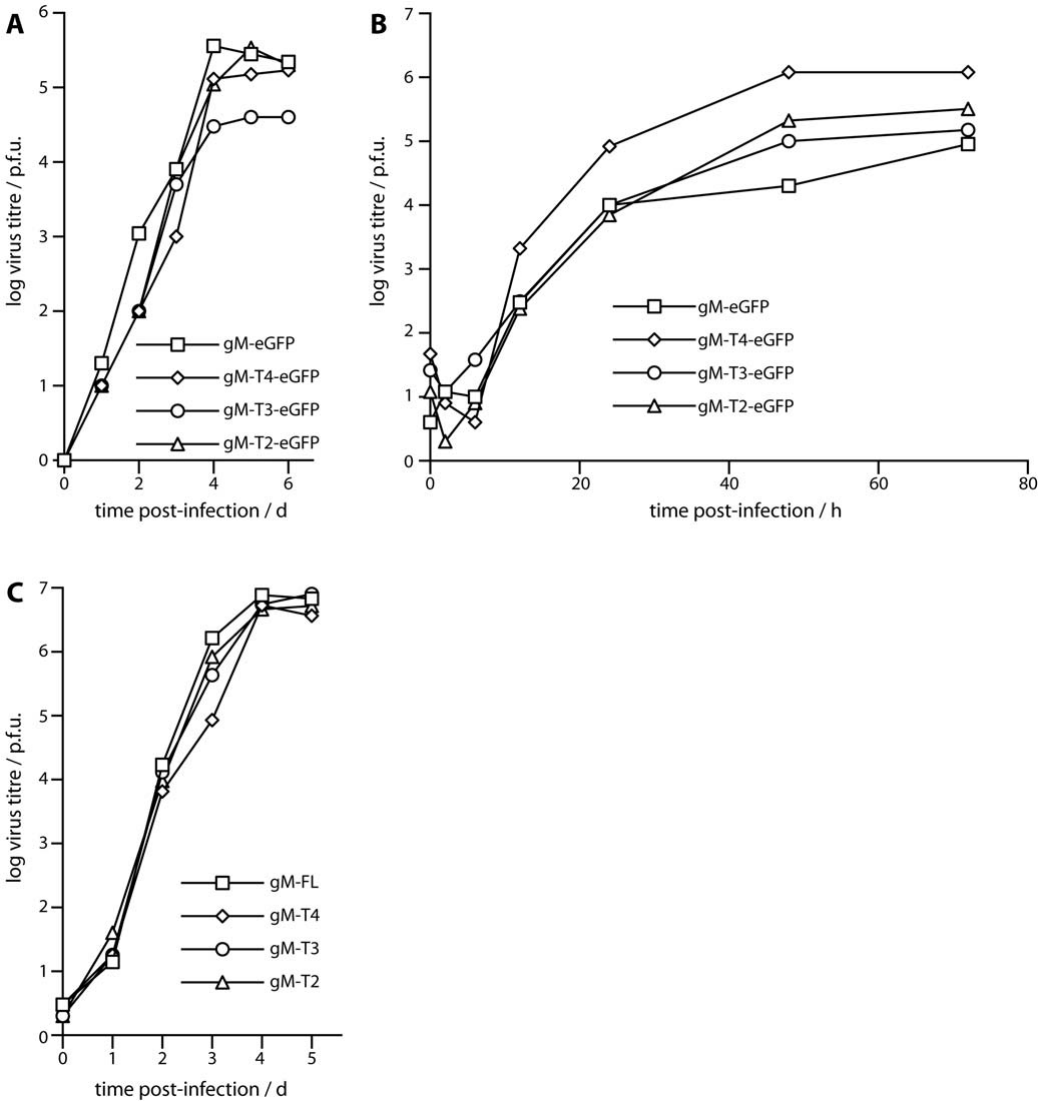
*In vitro* replication of the viable gM truncation mutants. A. BHK-21 cells were infected with full-length or truncated gM-eGFP viruses (0.01 p.f.u./cell). After 2 h at 37°C, unattached virus was removed by washing ×2 in PBS. The virus titres of replicate cultures were then determined by plaque assay at the times shown. B. BHK-21 cells were infected with the same viruses as in A, but at 5 p.f.u./cell. After 2 h at 37°C, the input virus was inactivated by acid washing (pH = 4 isotonic citrate buffer). Virus titers were then determined by plaque assay at the times shown. C. BHK-21 cells were infected at low multiplicity as in A, but using the viruses with stop codons separating their gM and eGFP coding sequences. Virus titers were then determined by plaque assay.

In order to determine whether this phenotype had marked functional consequences, we assayed the *in vitro* lytic replication of the T2, T3 and T4 mutants after low multiplicity inoculation (Fig. 6). The truncation mutants showed at most a very minor replication lag (Fig. 6A). No replication deficit was seen after high multiplicity infection (Fig. 6B), and low multiplicity infections using independently derived truncation mutants that lacked eGFP tags on gM were essentially normal (Fig. 6C). We concluded that there was no major deficit in *in vitro* lytic propagation with the T2, T3 or T4 truncations.

### *In vivo* growth of the viable gM truncation mutants

**Figure 7:**
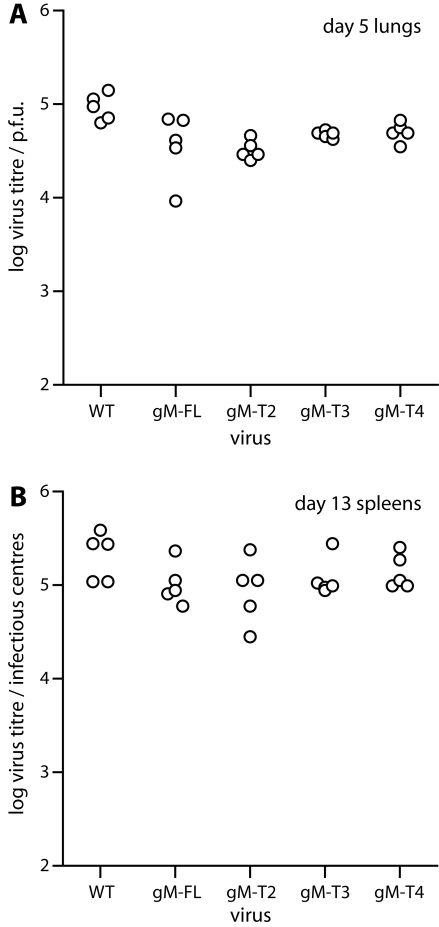
*In vivo* replication of the viable gM truncation mutants. A. C57BL/6 mice were infected intranasally (10^4^ p.f.u. in 30 µl) with wild-type (WT) or gM mutant viruses as shown. All these had their gM and eGFP coding sequences separated by stop codons, so gM was untagged. The infectious virus titres in lungs were determined by plaque assay at 5 days post-infection. Each point shows the titre per organ of one mouse. B. Mice were infected intranasally as in A, and 13 days later titered for splenic virus by infectious centre assay. This is an assay of reactivatable latent virus, since pre-formed infectious virus is essentially undetectable in lymphoid tissue after intranasal infection. Again each point corresponds to the titre per organ of one mouse.

We then tested the untagged T2, T3 and T4 gM truncation mutants for *in vivo* host colonization after intranasal inoculation (Fig. 7). All the mutants showed a slight deficit in lytic replication in infected lungs compared to wild-type MuHV-4 (Fig. 7A), but they showed no deficit compared to the full-length gM control (which included the same stop codons and downstream eGFP coding sequence). None of the truncation mutants showed a reduction in peak latent viral load in the spleen (Fig. 7B). We concluded that while eGFP tagging may have reduced lytic infection somewhat, perhaps because the virion tegument would have to incorporate multiple copies of eGFP, the T2–T4 gM truncations themselves had little or no effect on host colonization.

## Discussion

Our understanding of gamma-herpesvirus lytic functions has generally lagged behind that of the alpha- and beta-herpesviruses. This reflects that EBV and KSHV lytic functions are hard to study, and that the tumours these viruses cause are associated more with latent than with lytic infection. However, latency has proved a difficult therapeutic target, because few viral gene functions are expressed, and it is increasingly realized, particularly for KSHV [44], [45], that lytic and latent infections work together in host colonization, such that targetting one may help to control the other [46]. We are therefore using MuHV-4 as a template for working out how lytic functions contribute to gamma-herpesvirus replication and pathogenesis. Our aim here was to analyze specific gM functional domains. The non-viability of gN-deficient MuHV-4 [11] and the poor expression of gN without gM [12] imply that the gN-interacting gM extracellular loop [47] is essential. We therefore targetted another well-defined feature of gM, its cytoplasmic tail. The distal 31 amino acid residues of the 51 residue tail could be deleted with relative impunity, but while just 4 cytoplasmic tail residues were sufficient for gM expression, residues 5–20 were also required for function.

gM expressed without gN remained diffusely perinuclear, consistent with retention in the endoplasmic reticulum, even when all its cytoplasmic trafficking motifs were intact. Thus, gN is required for gM to reach its normal site of action in the trans-Golgi network [29]. This would explain why gN is essential for MuHV-4 replication. gM missing its acidic cluster and distal YXXΦ motif (gM-T2) was still re-localized by and co-localized with co-transfected gN, and replication of the corresponding virus mutant was accordingly preserved; but when the proximal YXXΦ motif of gM was also deleted (gM-T1), the expression of co-transfected gN was noticeably less, gM was not re-localized and the co-localization of gM and gN was much reduced. The corresponding mutant virus also failed to replicate. Thus, it appeared that the gM cytoplasmic tail is essential for a stable association with gN and therefore for MuHV-4 replication. gM lacking cytoplasmic trafficking motifs may not reach the right endoplasmic reticulum micro-domain to meet up with gN, or a subsequent failure of export may make the gM/gN complex unstable.

The phenotypes of our MuHV-4 gM mutants were entirely consistent with a recent *in vitro* analysis of the HCMV gM [48]. There, the acidic cluster was dispensible, and either the proximal or distal YxxΦ motif could be disrupted, but not both. Thus, while gM is dispensible for the replication of several alpha-herpesviruses, beta- and gamma-herpesviruses may be similar in their requirements for gM and its cytoplasmic tail. The redundancy of gM cytoplasmic trafficking motifs could possibly reflect an in-built robustness to maintain virion production despite DNA replication errors. But this would not explain why gM is so conserved - for example, gM was found to be identical in 6 otherwise diverse strains of murine CMV [49]. It seems more likely that single motif gM mutants have subtle defects that are important for evolutionary fitness, where a 10% difference in virus transmissibility would have a major impact, but hard to detect in experimental pathogenesis, where transmission is hard to reproduce and limits on animal numbers make replication reductions of <90% hard to demonstrate. The apparently dispensibility of gM for the replication of some alpha-herpesviruses [33], [36] may have a similar explanation: that gM-independent egress pathways make gM-dependent replication deficits difficult to define experimentally but still fail to off-set a powerful conservation of gM/gN in natural infection. The subtle change in capsid distribution with the T2–T4 mutants, and the failure to export a viable gM-T1/gN complex argued that the MuHV-4 gM cytoplasmic tail has interactions with the cellular transport machinery that play an important role in lytic replication.
